# Nitrogen Fertilizer Management in Dryland Wheat Cropping Systems

**DOI:** 10.3390/plants7010009

**Published:** 2018-01-29

**Authors:** Olga S. Walsh, Sanaz Shafian, Robin J. Christiaens

**Affiliations:** 1 Department of Plant Sciences, Southwest Research and Extension Center, University of Idaho, Parma, ID 83660, USA; sanazs@uidaho.edu; 2 Private Enterprise of Raths Ranch, Roundup, MT 59072, USA; idahoparma83660@gmail.com

**Keywords:** wheat, nitrogen, grain yield, protein content, nitrogen uptake

## Abstract

Wheat is the most widely cultivated food crop in the world, which provides nutrition to most of the world population and is well adapted to a wide range of environmental conditions. Timely and efficient rates of nitrogen (N) application are vital for increasing wheat grain yield and protein content, and maintaining environmental sustainability. The goal of this study was to investigate the effect of using different rates and split application of N on the performance of spring wheat in dryland cropping systems. The experiment was conducted in three different locations in Montana and Idaho during two consecutive growing seasons. A split-plot experimental design was used with three at planting N fertilization application (0, 90 and 135 kg N ha^−1^) and two topdressing N fertilization strategies as treatments. A number of variables such as grain yield (GY), protein content (GP) in the grains and N uptake (NUP) were assessed. There was a significant effect of climate, N rate, and time application on the wheat performance. The results showed that at-planting N fertilizer application of 90 kg N ha^−1^ has significantly increased GY, GP and NUP. On the other hand, for these site-years, increasing at-planting N fertilizer rate to 135 kg N ha^−1^ did not further enhance wheat GY, GP and NUP values. For all six site-years, topdress N fertilizer applied at flowering did not improve wheat GY, GP and NUP compared to at-planting fertilizer alone. As the risk of yield loss is minimal with split N application, from these results we concluded the best treatment for study is treatments that had received 90 kg N ha^−1^ split as 45 kg N ha^−1^ at planting and 45 kg N ha^−1^ at flowering.

## 1. Introduction

Achieving food security and improving nutrition have been listed as the mail of goals for sustainable development by United Nations [1]. By the middle of the 21st century, global food demand is expected to increase by 60% [2]. It is also estimated that 80% of this food production will have to be achieved by improving grain yield (GY) and crop intensification versus only 20% from expansion of arable land [3]. Therefore, the need for continuing productivity and maintained sustainability are the main concerns facing current agriculture [4].

Wheat is the most widely cultivated food crop in the world which provides nutrition (proteins, energy and minerals) to most of the world population [5,6], and is well adapted to a wide range of environmental conditions. Improving GY and quality are the main goals in wheat production. Wheat GY and protein content (GP) are highly dependent on genetic and environmental factors such as nitrogen (N) availability, water, temperature, and management practices [7,8] through their effects on GP and composition [9]. Among other inputs, N application is most effective for increasing wheat GY and GP content. Nitrogen fertilization also directly and radically impacts wheat production profitability. Hence, efficient N fertilizer management is critical for agronomic, economic, and environmental reasons [10,11]. 

Time and rates of N application are the most important factors of N fertilizer management. Failure to accurately estimate the appropriate rate and time for fertilizer application often results in inefficient fertilizer management [5,12]. Excessive N rates are often applied in the hopes of increasing wheat GY [13]. However, wheat GY increase is not linearly related to increases in N fertilizer rates. In addition, inefficient N fertilizer management has been linked to nitrate leaching, soil denitrification, ammonia volatilization, and nitrous oxide contamination of aquifers and aggravating the climate change [14,15]. 

Application of N fertilizer before or at the time of planting is considered most advantageous by some growers, as it helps to better allocate their time and labor [16] and benefit from superior soil conditions and lower fertilizer prices [17,18]. Mixed results were reported comparing preplant vs. split N fertilization. Bruns and Abbas [19] found that applying all N at or before planting should result in better economic returns. Preplant and at-planting fertilization has potential for minimizing N deficiency early in the growing season. On the other hand, applying all N in advance may result in N immobilization before plant uptake can take place [20,21]. Mid-season N fertilization enables matching the N supply with the crop’s N requirements, and provides vital nutrients at the time when N uptake (NUP) is at maximum, which facilitates efficient N fertilizer use [22]. Morris et al. [23] conducted an experiment to determine whether potential yield reductions from early-season N stress can be corrected using in-season N applications. They determined topdress N rate using a GreenSeeker hand-held sensor and an algorithm developed at Oklahoma State University. They reported that the application of N fertilizer to well-established wheat crop has resulted in the highest GYs. There are opportunities to benefit from premiums for high quality grain, when GP values fall below the market’s 14% threshold. Previous research has shown that topdress N applications at flowering could boost GP; it typically takes between 15–25 kg of N to increase wheat GP by 1% [24].

Wheat is an important staple food in U.S., ranking second behind corn in terms of planted acreage, production, and gross farm receipts [25]. Montana, with excellent growing condition, leads wheat producing states among the United States following North Dakota and Kansas [25]. Nitrogen is the nutrient most commonly limiting GY of spring wheat and other cereals in Montana [26]. Little is known about the effects of different combinations of N fertilizer rate and application time on spring wheat in semi-arid crowing conditions of Montana.

The objective of this study was to evaluate the effect of N rates and application times (all at-planting or split) on GY, GP content, and NUP of dryland hard red spring wheat grown in Montana semi-arid conditions. It was hypothesized that an appropriate combination of N rate and application time would maximize wheat GY and quality.

## 2. Results

**Figure 1:**
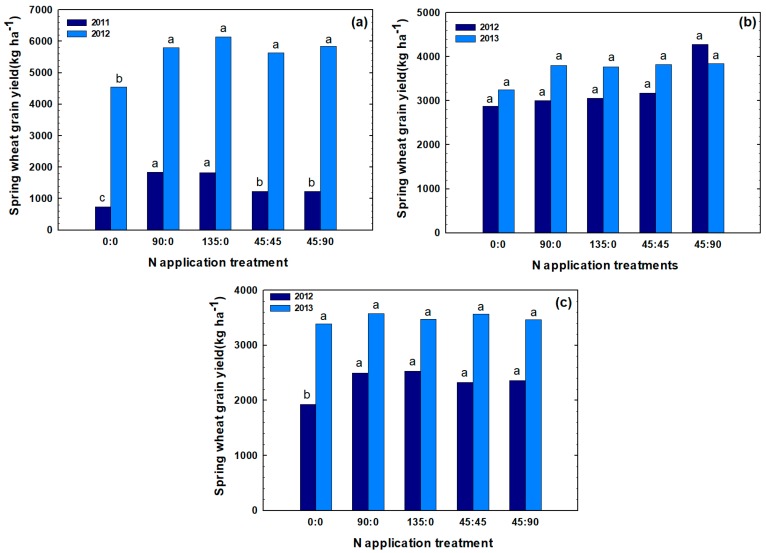
Effect of different nitrogen (N) rates and application time on spring wheat grain yield in Pondera (**a**), Chouteau (**b**), and Teton (**c**) in two years. Treatments were designated in the format x:y, where x and y are the fertilizer N rates in kg N ha^−1^ applied at planting and at flowering, respectively. Bars within the same year followed by the same letter are not significantly different (*p* > 0.05) based on a Duncan’s multiple range test.

Based on different N rates and application time, a wide range of wheat GY was obtained; GY ranged from 736 kg ha^−1^ to 6140 kg ha^−1^ (Figure 1). In Pondera, the highest mean GY of 6140 kg ha^−1^ was obtained in 2012 at a fertilizer N rate of 135 kg N ha^−1^ applied at planting time. In Chouteau, the maximum mean GY of 4275 kg ha^−1^ was obtained in 2012 when 45 kg N ha^−1^ at planting followed by 90 kg N ha^−1^ topdress was applied. In Teton, the highest mean GY of 3576 kg ha^−1^ was obtained in 2013 when 90 kg N ha^−1^ was applied at planting. Overall, GYs were notably higher in the second year for each location. Generally, observed variations in GY were due to differences in climate, inherent soil fertility, and N rate and application time.

When comparing combinations of N fertilizer rate and application time, in three site-years (Pondera 2011, 2012 and Teton 2012), the rate of at-planting N fertilizer significantly affected spring wheat GY. In these site-years, GYs were typically higher in plots that received 90 kg N ha^−1^ at planting time compared to check treatments. On the other hand, for these site-years, increasing at-planting N fertilizer rate to 135 kg N ha^−1^ did not result in significant differences in GYs (Figure 1 and Table 1). In the other three site years (Chouteau 2012, 2013 and Teton 2013), although at-planting N fertilizer application slightly increased GYs numerically, these differences were not statistically significant. For all six site-years, topdress N fertilizer applied at flowering did not improve wheat GYs, compared to at-planting fertilizer alone (Table 1). In four out of six site-years, the rate of total N fertilizer significantly affected spring wheat GYs. While GYs were typically higher in plots that received total of 90 kg N ha^−1^, increasing total N fertilizer rate to 135 kg N ha^−1^ did not further improve GY in any of the 6 site-years (Table 1).

**Figure 2:**
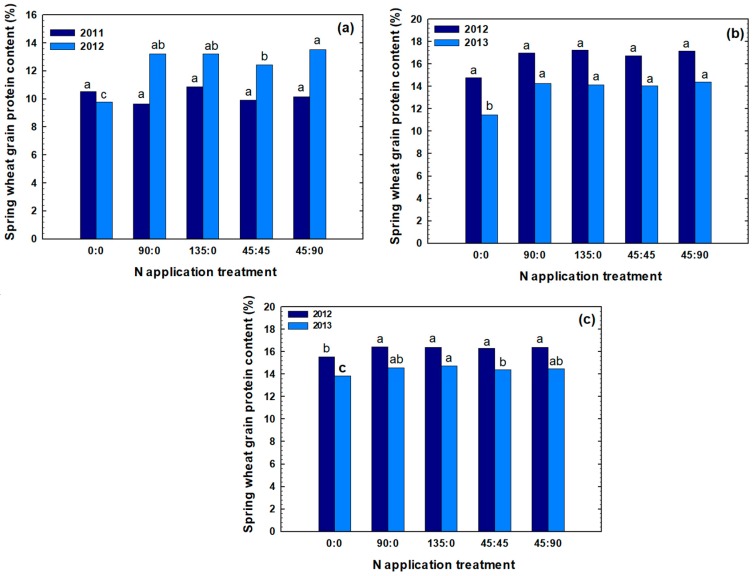
Effect of different rate and timings of N application on protein content (GP) of spring wheat in Pondera (**a**), Chouteau (**b**), and Teton (**c**) in two years. Treatments were designated in the format x:y where x and y are the fertilizer N rates in kg N ha^−1^ applied at planting and at flowering, respectively. Bars within the same year that have the same letter are not significantly different (*p* < 0.05) based on a Duncan’s multiple range test.

Similar to the grain yield, a wide range of GP ranging from 9.7% to 17.3% were obtained (Figure 2). Grain protein generally increased with increasing fertilizer N rate applied at planting. In Pondera, the highest mean GP of 13.5% was obtained in 2012 when 45 kg N ha^−1^ N was applied at planting followed by 95 kg N ha^−1^ at flowering time. In Chouteau, the maximum mean GP of 17.3% was obtained in 2012 when 135 kg N ha^−1^ was applied at planting. In Teton, the highest mean GP of 16.4% was obtained in 2012 when 90 kg N ha^−1^ was applied at planting or 45 kg N ha^−1^ was applied at planting followed by 90 kg N ha^−1^ was applied at flowering. Overall, GP were higher in the first year for each location (except for Pondera). In Pondera in 2011, N fertilizer rate or application time did not significantly affect GP. This was the lowest yielding site-year, and it is reasonable to suggest that most N taken up by the crop was used to produce GY, not GP. On the other hand, in Pondera in 2012, the higher yield potential situation, application of 90 or 135 kg N ha^−1^ at planting resulted in comparable protein values, but increasing topdress N rate from 45 to 90 kg N ha^−1^ has significantly improved GP (Table 1). In general, at all other site-years, at-planting N fertilizer application of 90 kg N ha^−1^ has significantly increased GP values. On the other hand, for these site-years, increasing at-planting N fertilizer rate to 135 kg N ha^−1^ did not further enhance wheat GP (Figure 2 and Table 1).

**Figure 3:**
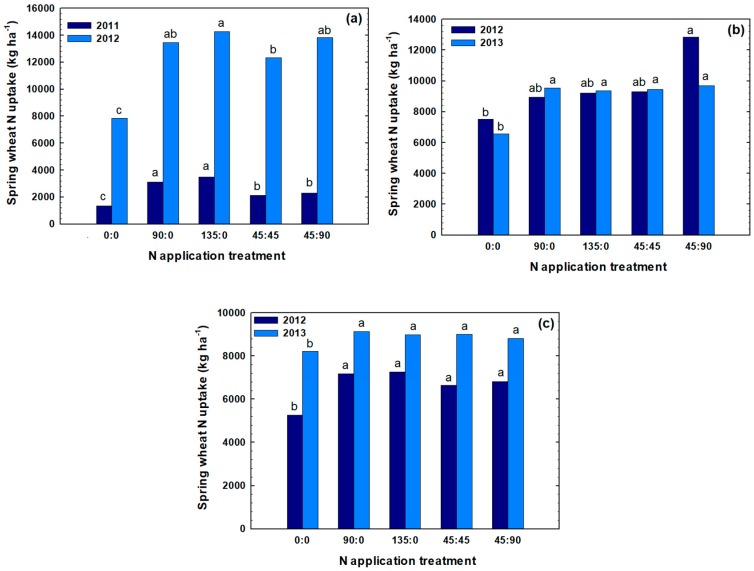
Effect of different rate and timings of N application on N uptake (NUP) of spring wheat in Pondera (**a**), Chouteau (**b**), and Teton(**c**) in two years. Treatments were designated in the format x:y where x and y are the fertilizer N rates in kg N ha^−1^ applied at planting and at flowering, respectively. Bars within the same year that have the same letter are not significantly different (*p* < 0.05) based on a Duncan’s multiple range test.

Similar to the GY and GP, spring wheat NUP at maturity was significantly affected by rate and time of N application across sites and years (Figure 3 and Table 1). Different NUP values ranging from 1350 to 14,261 kg ha^−1^ were obtained. In Pondera, in both 2011 and 2012, the highest NUPs were obtained when 135 kg N ha^−1^ was applied at planting. In Chouteau in 2012 and 2013, the highest NUP were obtained when 45 kg N ha^−1^ was applied at planting following by 90 kg N ha^−1^ at flowering. In Teton in 2012, the highest NUP was obtained when 135 kg N ha^−1^ was applied at planting, while in 2012 the highest NUP was obtained when 90 kg N ha^−1^ was applied at planting. In Pondera in 2011, similar to GY and GP, minimum NUP values may have resulted due to N losses via runoff and leaching, however N fertilizer rate or application time still had a significant effect on spring wheat NUP.

For all site-years, application of 90 kg N ha^−1^ significantly increased NUP values compared to unfertilized check, while increasing N rate from 90 to 135 kg N ha^−1^ at planting did not significantly increase NUP. In addition, there were no significant differences among 90 kg N ha^−1^ and 135 kg N ha^−1^ fertilizer rates applied as topdress. In three site-years (Chouteau 2013 and Teton 2012, 2013), there were no significant differences among applying N at planting or topdress (Figure 3). In Pondera for both years, N application as topdress significantly decreased N uptake. In Chouteau, in 2012, only topdress N application at 90 kg N ha^−1^ significantly increased NUP. These results suggest that all N applied at planting was taken up by wheat before flowering, and additional N supplied at flowering helped to boost both GY (substantial, but not statistically significant, Figure 1) and especially GP (Figure 2 and Table 1). These results are consistent with previous studies that showed that the late-season N fertilization was an effective measure in increasing GP content [27,28]. 

**Figure 4:**
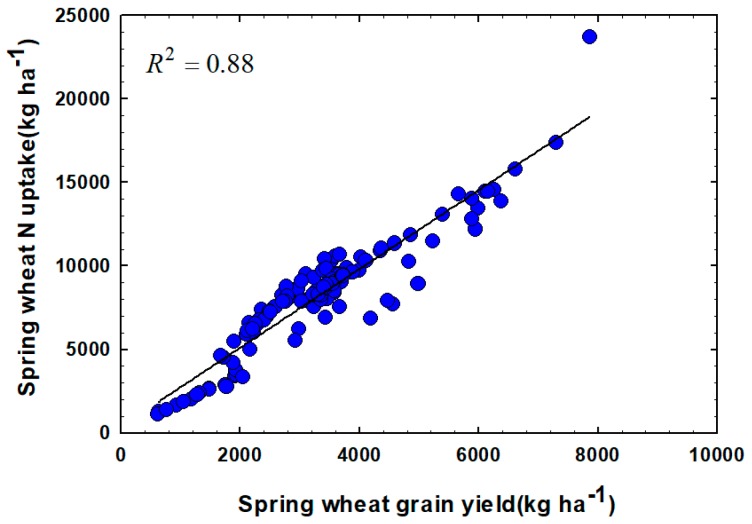
Relationship between N uptake and grain yield of spring wheat, six site-years in Montana.

The relationship between NUP and GY as affected by N treatments at all locations is illustrated in Figure 4. Overall, independently of the locations and years, there was a strong positive linear relationship between NUP and GY of the plant wheat with R^2^ = 0.88. This indicates that the N requirement per kg of GY increases at the higher yield levels which is consistent with reports from previous studies [29]. This relationship provides a measure of total N uptake by the crop for achieving a certain GY level, and can prove very useful in developing algorithms for improving N fertilizer management strategies.

## 3. Discussion

**Figure 5:**
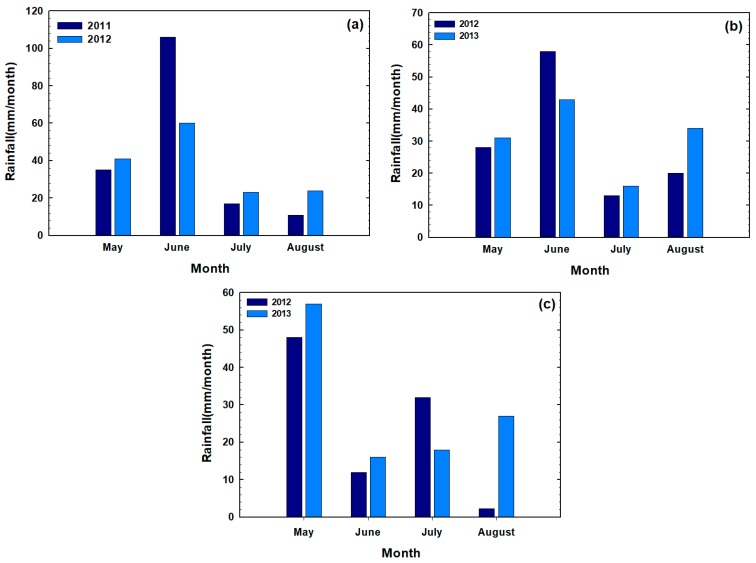
Monthly rainfall at Pondera (**a**), Chouteau (**b**), and Teton (**c**) during growing season.

In Pondera in 2011, the experiment was established in a re-cropped (continuous wheat) field, not a typical fallow-wheat rotation, which may have negatively impacted initial crop stand establishment due to lower soil moisture and residual soil N content. Furthermore, in 2011, Pondera received significant rainfall in June (Figure 5), which resulted in flooding and water standing in the fields for several days at the time. These extreme precipitation events within a short time period most likely affected GY. Wheat can withstand three to four days of flooding before GY is impacted negatively, granted the leaves remain above the water. However, in most cases, waterlogged conditions result in oxygen depletion and acute N deficiencies due to impaired NUP. Flooding has been linked to poor leaf elongation and lower kernel number. Ultimately, wheat GY can be decreased by 1 to 5% per each day the crop is flooded, especially when temperatures exceed 18 °C, with 20 to 50% GY loss occurring with waterlogged conditions for more than one week [30]. Waterlogged conditions could be the probable cause for the very low, non-typical GY for Pondera in 2011. 

Growing conditions were generally good in 2012, with timely, spread out throughout the growing season (May–August) rainfall (Figure 5). In 2012, Pondera received the highest amount of rainfall, following by Chouteau and Teton. Higher soil moisture content due to timely precipitation has resulted in higher wheat GY in Pondera and Chouteau, compared to Teton. Chouteau received similar amount of rainfall in 2012 and 2013, thus greater GY in 2013 was likely due to higher soil N content in 2013 (Table 2). At Teton, GY were higher in 2013 compared to 2012, corresponding to higher soil N content (26.8 kg N ha^−1^ in 2013 vs. 14.3 kg N ha^−1^ in 2012) and higher rainfall (118 mm vs. 94 mm). Understandably, a response to applied N at planting was greater in 2012, compared to hardly any response in 2013.

These results show that in a higher GY potential situation, when substantial response to applied N is expected (such as Pondera in 2012) the benefit of N fertilization is greater. On the other hand, in a lower yield potential environment, with lower potential response to N (like Pondera in 2011), increasing topdress rates N rates in hopes of optimizing GY, does not pay off.

In this study, there were no significant differences in GY, GP, or NUP among 90 kg N ha^−1^ and 135 kg N ha^−1^ fertilizer rates (except for GP and NUP at Pondera in 2012), and topdress N fertilizer applied at 45 kg N ha^−1^ significantly increased GP. Thus, in terms of GP production, the best treatment for most site-years was treatment 4: 45 kg N ha^−1^ at planting time followed by 45 kg N ha^−1^ at flowering (Figure 2). In this study, there was no benefit of split N application in terms of GY, GP or NUP. 

The only site-year, where split-applied 90 kg N ha^−1^ resulted in lower yields compared to the same amount applied at planting was Pondera, in 2011, where waterlogged conditions may have substantially depleted N in the soil, thus treatments that received only 45 kg N ha^−1^ at planting did not perform well. In the other five of six site-years, treatments that received a total of 90 kg N ha^−1^ at planting yielded similarly to those that received 90 kg N ha^−1^ split as 45 kg N ha^−1^ at planting and 45 kg N ha^−1^ at flowering. This suggests that supplying 50% of N as a starter has allowed adequate wheat stand establishment and was able to successfully carry the crop through the vegetative growth stages. Then, further addition of N at flowering helped to optimize GY and GP. At the same time, treatments receiving all N at planting performed as well as those receiving split N, suggesting that the N loss from the plant-soil soil system was minimal and N supplied at the time of planting has remained in the soil available for plant uptake throughout the growing season.

The period of most rapid NUP by wheat occurs during stem elongation, so application of all N prior to or at seeding is not advisable, especially in dryland wheat production systems, where crop yield potential and environmental conditions (e.g., the amount of precipitation the crop will receive) are not yet known. Providing just enough N to allow good crop stand establishment and then topdressing according to mid-season crop status evaluation, and taking into account environmental conditions is a more efficient approach to nutrient management. Not being able to accurately predict precipitation and temperature later in the season that might substantially affect GY and quality is still a big challenge. However, having a clear picture of growing conditions for the first part of the season and being able to evaluate crop’s nutrient status using tissue sampling, chlorophyll meters or precision sensors, will allow us to make more informed nutrient management decisions. 

## 4. Materials and Methods

### 4.1. Experimental Locations

Dryland experiments were established at Montana State University’s Western Triangle Agricultural Research Center, near Conrad, Pondera County, MT (48.309794, −111.924684) for 2 growing seasons in 2011 and 2012, and in collaborating grower’s fields in Chouteau County, MT (48.111104, −110.136420) and Teton County (43.811284, −111.223261) for the 2012 and 2013 growing seasons. Initial soil test results for each location are detailed in Table 2.

Hard red spring wheat (cv. Choteau) was planted with a small plot drill with Conserva Pak^TM^ openers manufactured by Swift Machining (Washougal, WA, USA) at a density of approximately 1.8 million plants ha^−1^. The experimental design was a randomized complete block design (RCBD) with four replications. The main plot treatments were granular urea (46-0-0) applied at planting time at four N rates (0, 45, 90 and 135 kg N ha^−1^). The subplot treatments were three urea topdress rates: 0, 45 and 90 kg N ha^−1^ applied at Feekes 10 (flowering) (Table 3). The top total application rate of 135 kg N ha^−1^ was chosen using the fertilizer guidelines for dryland hard red spring wheat grown in Montana (1.3 kg per each 67.2 kg of wheat grain) and the typical GY levels of about 2688 kg ha^−1^ for the region. Previous fertility work in the area has shown that current fertilizer guidelines may potentially be reduced (to 0.9 kg per each 67.2 kg of wheat grain) without resulting in GY or GP penalties. Based on that, the lower total application rate of 90 kg N ha^−1^ was chosen in this study.

Treatment 1 was used as unfertilized check plot to access wheat response to N at each site-year. At-planting urea was sidebanded about 1.90 cm to the side and about 2.54 cm above the seed to enhance wheat stand establishment. Individual plot size was 1.5 by 7.6 m. Topdress N application was done at Feekes 10 growth stage (flowering) by manually broadcasting urea without incorporation. 

### 4.2. Field Data Collection

A self-propelled Wintersteiger Classic combine (Wintersteiger Inc., Salt Lake City, UT, USA) was used to harvest hard red spring wheat at maturity. For each location, plot GY was recorded using a Harvest Master GrainGage, by Wintersteiger. The harvested wheat grain was dried in the oven at the temperature of 35 °C for 14 days; then, the dried samples were weighed to determine the accurate by-plot GY, which was adjusted to 12% moisture. From each by-plot sample, 400 g sub-sample was taken and analyzed by the Agvise Laboratories (Northwood, ND, USA) for total grain N content using near infrared reflectance spectroscopy (NIR) with a Perten DA 7250 NIR analyzer (Perten Instruments, Inc., Springfield, IL, USA). The DA 7250 is a proven, full-spectrum NIR instrument designed for use in the flour milling industry. It performs a multicomponent analysis in only 6 seconds using novel diode array technology which collect and average a large number of full spectra. As the sample is analyzed in an open dish, the problems associated with sample cups are avoided and operator influence on results is minimal. 

### 4.3. Statistical Analysis

Wheat GP content of spring wheat was calculated by multiplying grain N content by 5.7. Grain NUP was calculated by multiplying GY by total N concentration in the grain. Treatment effect on spring wheat GY, GP, and NUP were evaluated using the analysis of variance (ANOVA). ANOVA was conducted using the PROC GLM procedure in SAS v9.4 (SAS Institute, Inc., Cary, NC, USA) to detect any significant treatment effects. Orthogonal Contrasts method was used to separate means at a significance level of 0.05.

## 5. Conclusions

Timely and efficient use of N application can improve wheat GY and quality. In general, at-planting N fertilizer application of 90 kg N ha^−1^ significantly increased GY, GP and NUP values while increasing the rate to 135 kg N ha^−1^ did not further enhance wheat production. Similarly, increasing topdress N application rate from 90 to 135 kg N ha^−1^ did not significantly improve wheat performance. This indicates that 90 kg N ha^−1^ rate was adequate to optimize spring wheat GY and GP. As discussed above, the recommended N fertilizer application rate at the time this study was conducted was 1.3 kg per each 67.2 kg of wheat grain, with a typical GY of 2688 kg ha^−1^ for the area. In our study, with a very wide range of GY from 736 kg ha^−1^ to 6140 kg ha^−1^ (3438 kg ha^−1^ average across 6 site-years), wheat grain production was optimized with an application of 0.8 kg per 67.2 kg of wheat grain. This is consistent with our previous results, and suggests that current fertilizer guidelines may potentially be reduced without negatively affecting GY or GP, especially with new more productive, adaptable and efficient wheat varieties being developed for Montana region. Furthermore, we need to take into account that probably even less N is required to optimize wheat GY in areas with lower yield potential by satisfying crops need for N while also minimizing the potential for N loss from the soil-plant system. 

In addition, there were no significant differences in wheat production associated with N application time (at-planting vs. split). Although topdress N application did not significantly improve in wheat production, application of all N prior to or at seeding is not advisable, especially in dryland wheat production systems, where crop’s yield potential and environmental conditions are not yet known. Based on results of this study, application of 45 kg N ha^−1^ at planting followed by 45 kg N ha^−1^ at flowering could be recommended for dryland spring wheat cropping systems of Montana. 

## Figures and Tables

**Table 1:** Effect of at-planting N rate and topdress N rate on spring wheat grain yield (GY) and grain protein content (GP) for six site-years in Montana.

Effects	Pondera						Chouteau						Teton					
	2011			2012			2012			2013			2012			2013		
	GY, kg ha^−1^	GP, %	NUP, kg ha^−1^	GY, kg ha^−1^	GP, %	NUP, kg ha^−1^	GY, kg ha^−1^	GP, %	NUP, kg ha^−1^	GY, kg ha^−1^	GP, %	NUP, kg ha^−1^	GY, kg ha^−1^	GP, %	NUP, kg ha^−1^	GY, kg ha^−1^	GP, %	NUP, kg ha^−1^
	**At-Planting N Rate**																	
0 vs. 90	***	ns	***	**	***	***	ns	*	ns	ns	***	*	***	**	***	ns	***	*
0 vs. 135	***	ns	***	**	***	***	ns	*	ns	ns	***	*	***	**	***	ns	***	*
90 vs. 135	ns	ns	ns	ns	ns	ns	ns	ns	ns	ns	ns	ns	ns	ns	ns	ns	ns	ns
	**Topdress N Rate**																	
0 vs. 45	**	ns	**	**	***	***	ns	*	ns	ns	***	*	**	**	**	ns	***	*
0 vs. 90	**	ns	**	**	***	***	ns	*	*	ns	***	*	**	**	**	ns	**	*
45 vs. 90	ns	ns	ns	ns	**	ns	ns	ns	ns	ns	ns	ns	ns	ns	ns	ns	ns	ns
	**Total N Rate**																	
0 vs. 90	***	ns	**	**	***	***	ns	*	ns	ns	***	*	**	***	***	*	***	*
0 vs. 135	***	ns	***	**	***	***	ns	*	ns	ns	***	*	**	***	***	ns	***	*
90 vs. 135	ns	ns	ns	ns	*	*	ns	ns	ns	ns	ns	ns	ns	ns	ns	ns	ns	ns

*,**,*** Significant at the 0.05, 0.01, and 0.001 probability levels, respectively; ns is not significant.

**Table 2:** Preplant soil test results (0–60 cm) for six site-years in Montana.

	Pondera, 2011	Pondera, 2012	Chouteau, 2012	Teton, 2012	Chouteau, 2013	Teton, 2013
Total N (kg ha^−1^)	15.1	17.9	14.4	14.3	19.3	26.8
P (ppm)	23	23	20	17	15	21
K (ppm)	423	423	272	287	288	361
Organic Matter, %	2.9	2.9	3.7	2.6	3.1	2.7

**Table 3:** Treatment, at-planting N rate, topdress N rate, and total N rate applied at six site-years in Montana.

Fertilizer N Application			
Treatment	At-Planing Rate, kg N ha^−1^	Todress Rate, kg N ha^−1^	Total N Applied, kg N ha^−1^
1	0	0	0
2	90	0	90
3	135	0	135
4	45	45	90
5	45	90	135
